# American Dentists Abroad

**Published:** 1886-05

**Authors:** 


AMERICAN DENTISTS ABROAD.—Consul-General Raine, at Berlin, informs the Department of State that the Prussian minister of the interior and medicinal affairs has recently issued an order whereby dentists who have graduated in the United States are prohibited from practicing as "royally licensed" dentists. They can procure, however, a regular "trader's license," and practice under the same. They are prohibited entirely from establishing or opening dental dispensaries, (clinical institutions,) where practical dentistry is taught, but may receive patients for treatment. Licensed American dentists may be allowed to give instruction in technical dentistry, but are prohibited from lecturing and teaching dental surgery generally, or to give their places the character of a medical school, unless previously authorized to do so by the government.

A correspondence some years ago with the Hon. James G. Blaine, the Secretary of State, in regard to the status of diplomas from reputable American Dental Schools, revealed the fact that there is a disposition on the part of the German Officials to discountenance the practice of German students leaving their own country for better educational advantages. This action of the Prussian Minister of State will not, however, affect the standing of American educated dentists who may locate in Germany, as naturally their operations must in the end influence such practice, and maintain the high reputation in which the well educated practitioner is held throughout Europe.

